# Antibacterial activity against β- lactamase producing Methicillin and Ampicillin-resistants *Staphylococcus aureus*: fractional Inhibitory Concentration Index (FICI) determination

**DOI:** 10.1186/1476-0711-11-18

**Published:** 2012-06-20

**Authors:** Kiessoun Konaté, Jacques François Mavoungou, Alexis Nicaise Lepengué, Raïssa RR Aworet-Samseny, Adama Hilou, Alain Souza, Mamoudou H Dicko, Bertrand M’Batchi

**Affiliations:** 1 Laboratoire de Biochimie et Chimie Appliquées (LABIOCA), UFR/SVT, Université de Ouagadougou, Ouagadougou 09, 09 BP 848, Burkina Faso; 2 Institut de Recherche en Ecologie Tropicale (IRET/CENAREST), BP:13354, Libreville, Gabon; 3 Laboratoire de Phytopathologie, UFR Agrobiologie, Faculté des Sciences, Université des Sciences et Techniques de Masuku, Franceville, BP 943, Franceville, Gabon; 4 Institut de Pharmacopée et de Médecine Traditionnelle (IPHAMETRA), Centre National de la Recherche Scientifique et Technologique (CENAREST), BP:1156, Libreville, Gabon; 5 Laboratoire de Physiologie Animale, Electrophysiologie et de Pharmacologie, Faculté des Sciences, Université des Sciences et Techniques de Masuku, Franceville, Gabon; 6 Laboratoire de Biochimie Alimentaire, Enzymologie, Biotechnologie et Bioinformatique, UFR/SVT, Université de Ouagadougou, 09 BP 848, Ouagadougou 09, Burkina Faso

## Abstract

**Background:**

The present study reports the antibacterial capacity of alkaloid compounds in combination with Methicillin and Ampicillin-resistants bacteria isolated from clinical samples. The resistance of different bacteria strains to the current antibacterial agents, their toxicity and the cost of the treatment have led to the development of natural products against the bacteria resistant infections when applied in combination with conventional antimicrobial drugs.

**Method:**

The antibacterial assays in this study were performed by using inhibition zone diameters, MIC, MBC methods, the time-kill assay and the Fractional Inhibitory Concentration Index (FICI) determination. On the whole, fifteen Gram-positive bacterial strains (MRSA/ARSA) were used. Negative control was prepared using discs impregnated with 10 % DMSO in water and commercially available Methicillin and Ampicillin from Alkom Laboratories LTD were used as positive reference standards for all bacterial strains.

**Results:**

We noticed that the highest activities were founded with the combination of alkaloid compounds and conventional antibiotics against all bacteria strains. Then, results showed that after 7 h exposition there was no viable microorganism in the initial inoculums.

**Conclusion:**

The results of this study showed that alkaloid compounds in combination with conventional antibiotics (Methicillin, Ampicillin) exhibited antimicrobial effects against microorganisms tested. These results validate the ethno-botanical use of *Cienfuegosia digitata* Cav. (Malvaceae) in Burkina Faso. Moreover, this study demonstrates the potential of this herbaceous as a source of antibacterial agent that could be effectively used for future health care purposes.

## Background

Infectious diseases constitute the leading cause of premature deaths in the world and kill almost 50,000 people every day. An increase in antibiotic resistant bacteria is threatening world population with the recurrence of infectious diseases that were once thought to be under control at least in developed countries. In the recent years incidence of multi-drug resistance in Gram-positive, Gram-negative and other bacteria like *Mycobacterium tuberculosis* has been reported from all over the world [1]. These multi-drug resistant bacteria have also created additional problems in cancer and AIDS patients.

Methicillin resistant *Staphylococcus aureus* (MRSA) has gained much attention in the last decade, as the MRSA is a major cause of hospital acquired (nosoconical infections) β-lactam antibiotics are the preferred drugs against *Staphylococcus aureus* infections. *S.aureus* has developed resistance to the β-lactam antibiotics due to the production of chromosomal or plasmid mediated β-lactamases [2]. Moreover, increase incidence of vancomycin-resistant has also been reported [3]. Thus, the number of effective exogenous antibiotics is decreasing; therefore concerted efforts are to be made to identify antimicrobial materials from natural products and traditional medicines.

In effect, different extracts from traditional medicinal plants have been tested to identify the sources of the therapeutics effect [4]. Over the past 20 years, there has been an increased interest in the development of resistance of pathogens against antibiotics caused by the indiscriminate use of modern antibiotics [5,6]. As a result some natural products have been approved as new antibacterial drugs but there is still an urgent need to identify novel substances that are active towards pathogens with high resistance [7]. Considering the high cost of the synthetic drugs and their side effects, wide varieties of natural plants can be considered as a vital source for anti-microbial agents [8].

Therefore, the need of new and effective anti-microbial agents with broad-spectrum of activity from natural sources is increasing day by day [9]. In spite of the great advances observed in modern medicine therefore, plants still make an important contribution to health care. This is due in part to the recognition of the value of traditional medical systems and the identification of medicinal plants from indigenous pharmacopoeias which have significant healing power [10]. In addition, natural plant products, accordingly provide a contimal inspiration of bioactive antimicrobial agents with low toxicity, a broad spectrum and good pharmacokinetics to be clinically used without chemical modification [11]. So, such plants should be investigated to better understand their therapeutic properties, safety and efficiency [12].

Herbal medicine has become an ideal remedy for treatment of the diseases due to lesser amount of side effects, better compatibility and only accessible treatment for some diseases [13]. In the continuous search for active phytochemicals against pathogenic infectious *Cienfuegosia digitata* Cav. (Malvaceae) an herbaceous, has received considerable attention. This plant is a savannah herbaceous belonging to the family of Malvaceae and is abundantly distributed in the central and north of in Burkina Faso. Medicinally, it is used to heal the infectious wounds, to cure the cough, throat complaints, dysentery and others infectious diseases in children: diarrhoea, acute colitis, malaria, fever, pain, variola. In addition, this plant has antibacterial, anti-inflammatory, analgesic and hepatoprotective properties [14]. In previous study, the aqueous acetone extract from this herbaceous was evaluated for its antioxidant and anti-inflammatory activities [15] due to its composition in saponosides, coumarins, steroids, polyphenol, alkaloid compounds [14]. However, no systematic study has been reported concerning antibacterial activity of aqueous acetone extract of *Cienfuegosia digitata* Cav. This plant has not exhaustively been screened for antimicrobial activity.

Hence, the present study was conducted to study out the antibacterial of alkaloid compounds from *Cienfuegosia digitata* Cav., against the locally isolated microorganisms from patients having infectious diseases. The bacteriostatic, bactericidal, time-kill assay and Fractional Inhibitory Concentration Index studies of the alkaloid compounds were screened against clinical strains of bacteria with an aim of manufacturing some drugs like alkaloid compounds or a combination with conventional antimicrobial drugs to better manage resistant bacteria infectious diseases.

## Materials

### Plant materials

*Cienfuegosia digitata* Cav. (Malvaceae) was freshly collected in August 2008 in Gampela, 25 Km east of Ouagadougou, capital of Burkina Faso. The plant was botanically identified by Prof. Millogo-Rasolodimby from the plant Biology Department of the University of Ouagadougou. Voucher specimen (ID-10472) was deposited at the Herbarium of the “Laboratoire de Biologie et d’Ecologie Végétale, UFR/SVT of University of Ouagadougou”.

### Bacterial strains and antibiotics

Microorganisms used in this study were isolated from clinical samples at Laboratory of the General Hospital of Ouagadougou in Burkina Faso. Methicillin (25 μg) and Ampicillin (25 μg) were purchased from Alkom Laboratories LTD. Clinical isolates were: MRSA/ARSA-1, MRSA/ARSA-2, MRSA/ARSA-3, MRSA/ARSA-4, MRSA/ARSA-5, MRSA/ARSA-6, MRSA/ARSA-7, MRSA/ARSA-8, MRSA/ARSA-9, MRSA/ARSA-10, MRSA/ARSA-11, MRSA/ARSA-12, MRSA/ARSA-13, MRSA/ARSA-14 and MRSA/ARSA-15. The following microorganisms all identified by the use of their biochemical profiles as recommended by the manual “Bactériologie Medical” [16].

### Chemicals

All reagents were of analytical grade. Acetone, n-hexane were supplied by Fluka chemie (Buchs, Switzerland). INT (p-iodonitrotetrazolium chloride) was purchased from sigma-Aldrich chemie (Steinheim, Germany).

## Methods

### Preparation of alkaloid compounds

The harvested plant materials fresh (broken into leaf stems) were dried in the laboratory at room temperature (20-25 °C), afterwards samples were ground and made alkaline and 50 g were used with 28 % ammonia and extracted with chloroform at room temperature for a total period of 24 h and then the extract was partitioned between 5% HCl and Chloroform. The aqueous phase was made alkaline again with ammonia and partitioned between water and chloroform. Finally chloroform was totally evaporated from the organic phase to form the alkaloids powder [17].

### *In vitro* antibacterial activity

#### Preparation of inocula

The susceptibility tests were performed by Mueller Hinton agar-well diffusion method [18]. The bacterial strains grown on nutrient agar at 37 °C for 18 h were suspended in a saline solution (0.9 %, w/v) NaCl and adjusted to a turbidity of 0.5 Mac Farland standard (10^8^ CFU/ml). To obtain the inocula, these suspensions were diluted 100 times in Muller Hinton broth to give 10^6^ colony forming units (CFU)/ml [19].

#### Preparation of discs

The stock solutions of alkaloid compounds from *Cienfuegosia digitata* Cav. (Malvaceae) was dissolved in 10 % dimethylsulfoxide (DMSO) in water [20] at a final concentration of 400 μg/ml. The stock solution of alkaloid compounds from *Cienfuegosia digitata* Cav. (Malvaceae) was sterilized by filtration through 0.22 μm sterilizing Millipore express filter. The sterile discs (6 mm) were impregnated with 10 μL of the sterile alkaloid compounds from *Cienfuegosia digitata* Cav. Negative controls were prepared using discs impregnated with 10 % DMSO in water and commercially available antibiotic diffusion discs (Methicillin 25 μg and Ampicillin 25 μg from Alkom Laboratories LTD) were used as positive reference standards for all bacterial strains.

#### Disc-diffusion assay

Petri plates (9 cm) were prepared with 20 ml of a base layer of molten Mueller Hinton agar (DIFCO, Becton Dickinson, USA). Each Petri plate was inoculated with 15 μl of each bacterial suspension (10^6^ CFU/ml). After drying in a sterile hood, 6 mm diameter discs soaked with 10 μl of the different alkaloid compounds from *Cienfuegosia digitata* Cav. (Malvaceae) dilutions were placed on the agar. Discs containing Methicillin (25 μg) and Ampicillin (25 μg) were used as positive controls and 10 % DMSO was used as a negative control. The plates were incubated for 24 h at 37 °C. The diameters of the inhibition zones were evaluated in millimeters. The extract inducing inhibition zone ≥ 3 mm around disc were considered as antibacterial. All tests were performed in triplicate and the bacterial activity was expressed as the mean of inhibition diameters (mm) produced [21].

#### Micro-well dilution assay

Minimum inhibitory concentration (MIC) was determined by the microdilution method in culture broth as recommended previously [22]. Eight serial two-fold dilutions of alkaloid compounds or conventional antibiotics were prepared as described before, to obtain final concentration range of 400 to 3.125 μg/ml. The 96-well micro-plates (NUNC, Danemark) containing 100 μL of Mueller Hinton (MH) broth were used. For each bacteria strain, three columns of eight wells to the micro-plate were used. Each well has getting: the culture medium + alkaloid compounds or Methicillin or Ampicillin or the combination of alkaloid compounds with Methicillin or Ampicillin + inoculum (10 μl of inocula) and INT (50 μl; 0.2 mg/ml). The plates were covered and incubated at 37 °C for 24 h. All tests were performed in triplicate and the bacterial activity was expressed as the mean of inhibitions produced. Inhibition of bacterial growth was judged by rose or yellow colour. The MIC was defined as the lowest concentration of the alkaloid compound rich-fractions at which no colony was observed after incubation. So, the MIC was defined as the lowest concentration at which no visible growth was observed.

#### Minimal bactericidal concentration (MBC)

Minimum bactericidal concentration (MBC) was recorded as a lowest extract concentration killing 99.9 % of the bacterial inocula after 24 h incubation at 37 °C. Each experiment was repeated at least three times. MBC values were determined by removing 100 μl of bacterial suspension from subculture demonstrating no visible growth and inoculating nutrient agar plates. Plates were incubated at 37 °C for a total period of 24 h. The MBC is determined with the wells whose the concentrations are ≥ MIC [21,23].

#### Evaluation of bactericidal and bacteriostatic capacity

The action of an antibacterial on the bacterial strains can be characterized with two parameters such as Minimum inhibitory concentration (MIC) and Minimum bactericidal concentration (MBC). According to the ratio MBC/MIC, we appreciated antibacterial activity. If the ratio MBC/MIC = 1 or 2, the effect was considered as bactericidal but if the ratio MBC/MIC = 4 or 16, the effect was defined as bacteriostatic [24].

#### Time-kill assay

A bactericidal effect is defined as a 3 Log decrease in the CFU/ml or a 99.9 % kill over a specified time [25]. The definition of kill for this study has been used as per [20]. Kill-time can be determined at 6 h [26]. A 90 % kill at 6 h is equivalent to a 99.9 % kill at 24 h [27]. In this study the kill measurement was determined by the actual reduction in viable counts at 6 h for each isolate. Bacteria strains possessing the bactericidal effect were chosen to perform time-kill assay. Thus, 0.5 Mac Farland standards suspensions of microorganisms were diluted to have 50 ml of approximately 10^6^ CFU/ml in nutriment broth, and the concentration corresponding to the best MIC, were respectively added to the corresponding culture. The cultures were incubated at 37 °C. At 0, 1, 2, 3, 4, 5 and 6 h, an aliquot of 100 μl was removed and diluted with 10 ml sterile broth. The obtained suspension was used to inoculate 9 cm diameter Petri plates with a sterile non toxic cotton swab on a wooden applicator as indicated before in the agar-well diffusion assay. After 24 h incubation at 37 °C, the viability of microorganisms was evaluated by the presence of colonies on the plates and the experiment was carried out twice.

#### Evaluation of the fractional inhibitory concentration index of alkaloid extracts

The Muller Hinton agar dilution method was used to evaluate the Fractional Inhibitory Concentration Index (FICI) of alkaloid compounds from *Cienfuegosia digitata* Cav. (Malvaceae) and the tested anti-microbial standards as reported earlier [28,29]. Eight serial two-fold dilutions of alkaloid compounds were prepared as described before, to obtain final concentration range of 400 to 3.125 μg/ml. A series of two-fold serial dilutions of Methicillin or Ampicillin was also prepared in the same conditions as alkaloid compounds. In this way, all antibacterial standards dilutions were mixed with the appropriate concentration of alkaloid compounds thus obtaining a series of the combinations of conventional antibiotics and alkaloid compounds. The concentrations prepared corresponded to 1; 1/2; 1/4; 1/8; 1/16; 1/32; 1/64; 1/128; 1/256 of MIC values. The 96-well micro-plate (NUNC, Danemark) containing 100μL of Mueller Hinton (MH) broth were used. For each bacteria strain, three columns of eight wells to the micro-plate were used. Each well has getting: the culture medium + combination of alkaloid compounds with Methicillin or Ampicillin + inoculum (10 μl of inocula) and INT (50 μl; 0.2 mg/ml). The plates were covered and incubated at 37 °C for 24 h. All tests were performed in triplicate and the bacterial activity was expressed as the mean of inhibitions produced. Inhibition of bacterial growth was judged by rose or yellow colour. The analysis of the combination of alkaloid compounds and Methicillin or Ampicillin was obtained by calculating the Fractional Inhibitory Concentration Index (FICI) as follows: FICI = (MIC of the combination of alkaloid compounds and Methicillin or Ampicillin/MICa alone) + (MIC of the combination of alkaloid compounds and Methicillin or Ampicillin/MICb alone), where MICa (Minimal Inhibitory Concentration of alkaloid compounds from *Cienfuegosia digitata* cav.) and MICb (Minimal Inhibitory Concentration of Methicillin or Ampicillin). The FICI was interpreted as follows: (1) a synergistic effect when FICI ≤ 0.5; (2) an additive or indifferent effect when FICI >0.5 and <1 and (3) an antagonistic effect when FICI >1. This study was carried out following [30] method with light modifications.

## Results

**Figure 1:**
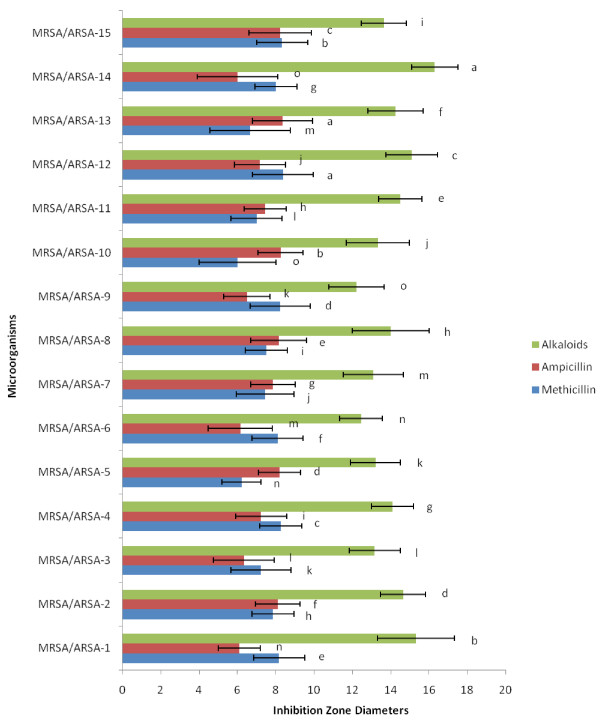
** Inhibition Zone Diameters of Alkaloid compounds from*Cienfuegosia digitata*Cav. and conventional antibiotics (Methicillin and Ampicillin).** The results are the means of number of the colonies ± standard deviations.

**Figure 2:**
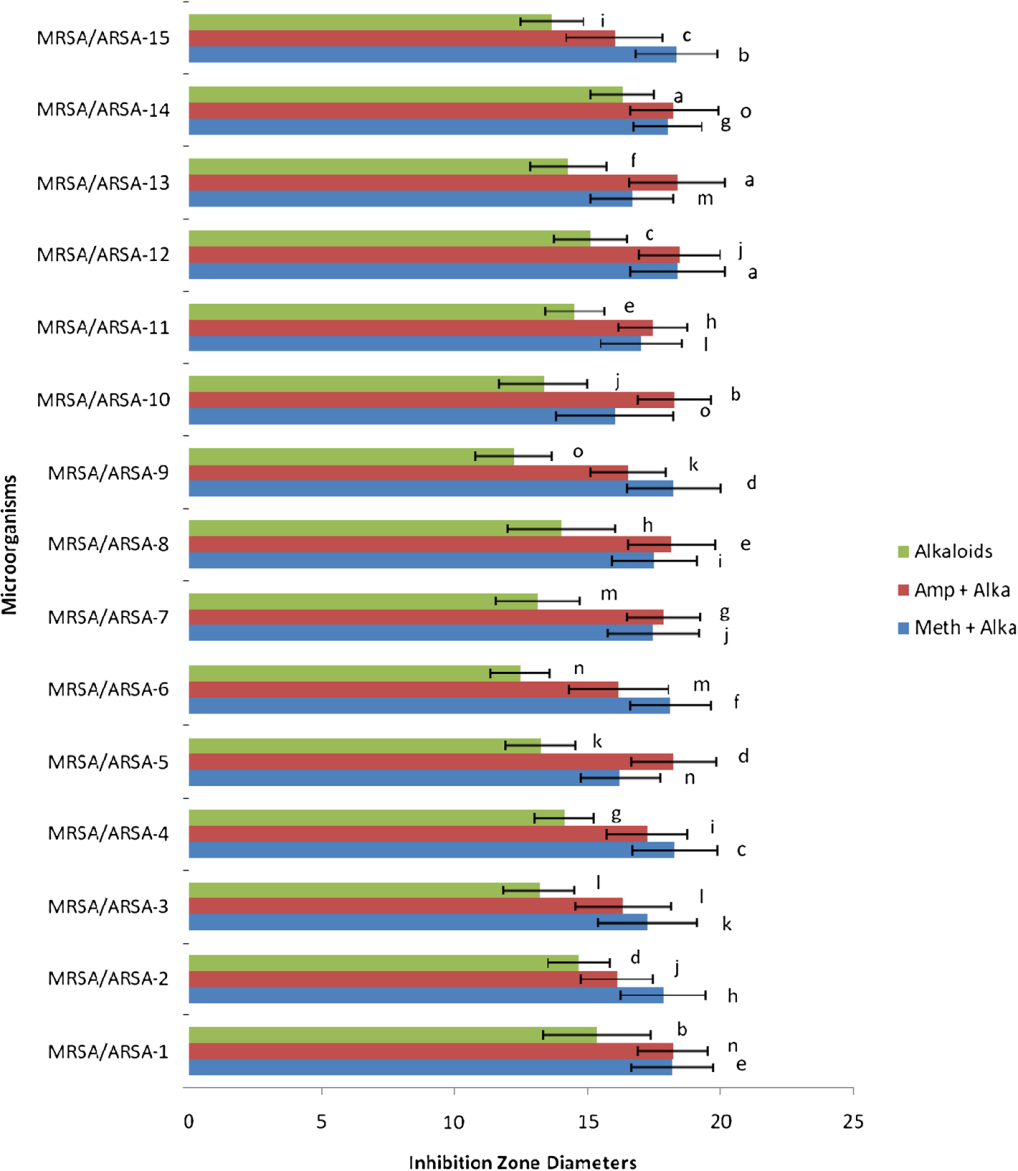
** Inhibition Zone Diameters of Alkaloid compounds and their combination with conventional antibiotics (Methicillin and Ampicillin).** Amp= Ampicillin; Meth= Methicillin; Alk= Alkaloids. The results are the means of number of the colonies ± standard deviations.

The present study reports the antibacterial potency of alkaloid compounds and conventional antibiotics. Alkaloid compounds and conventional antibiotics were tested for antibacterial activity against fifteen clinical isolates of Gram-positive bacteria (MRSA/ARSA) by disc diffusions, Minimum Inhibitory Concentration (MIC), Minimum bactericidal concentration (MBC), Time-kill assay and Fractional Inhibitory Concentration Index (FICI). One noticed that the susceptibility of the bacteria to the alkaloid compounds and in combination with conventional antibiotics on the basis of inhibition zone diameters varied according to microorganism, the results are reported in (Figure 1 and 2). There is a significant variation in the diameters of inhibition zone values (DIZ) of alkaloid compounds and their combination with conventional antibiotics. One noticed that all bacterial strains are resistant to the antibiotics comparatively to alkaloid compounds (Figure 1 and 2).

**Figure 3:**
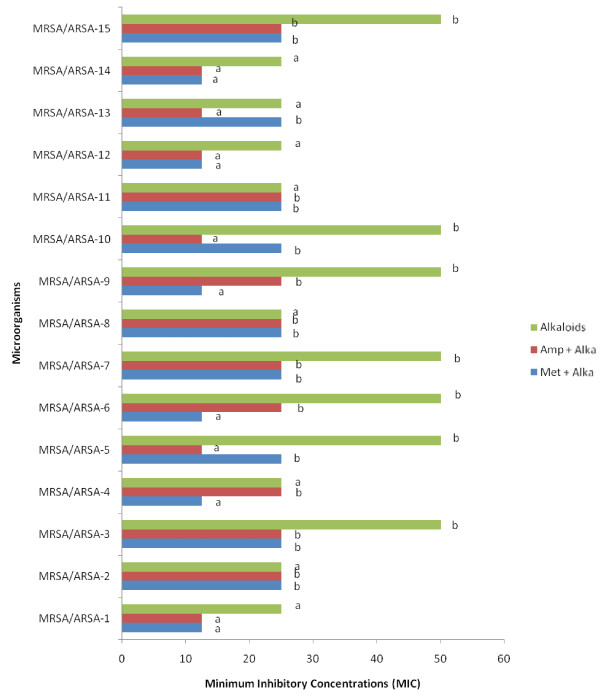
** Minimum Inhibitory Concentration (MIC) of alkaloid compounds from*Cienfuegosia digitata*Cav. and their combination with Methicillin and with Ampicillin.** The results are the means of number of the colonies ± standard deviations.

**Figure 4:**
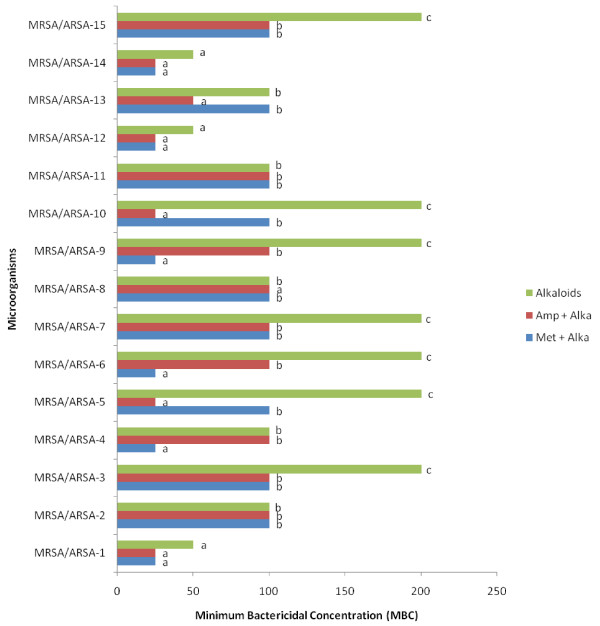
** Minimum bactericidal Concentration (MBC) of alkaloid compounds from*Cienfuegosia digitata*Cav. and their combination with Methicillin and with Ampicillin.** The results are the means of number of the colonies ± standard deviations.

**Figure 5:**
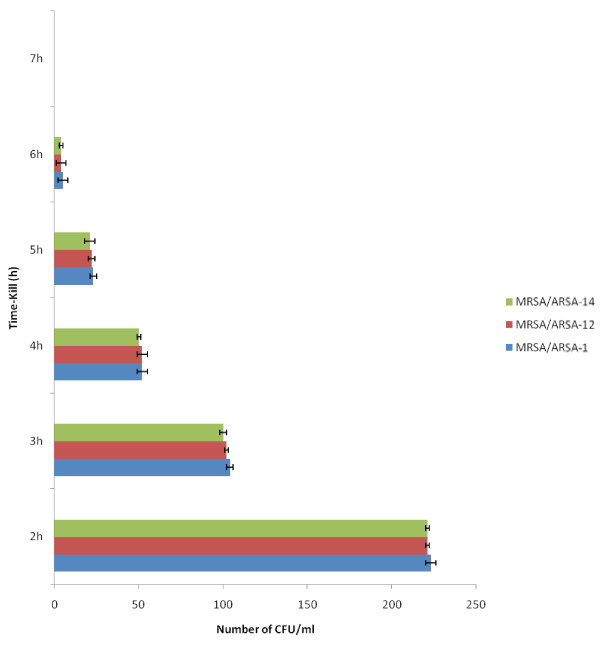
** Viability of microorganisms after 6 hours exposure with Alkaloid compounds from*Cienfuegosia digitata*Cav.** Absence of colonies after 6 h. CFU uncountable before 2 h. The results are the means of number of the colonies ± standard deviations.

**Figure 6:**
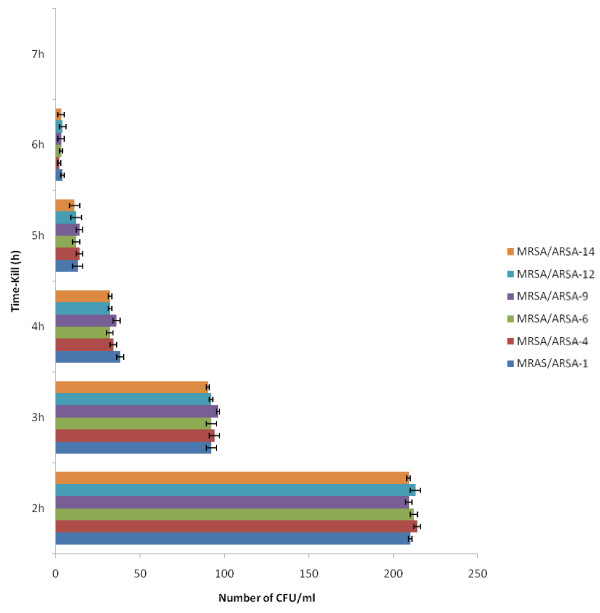
** Viability of microorganisms after 6 hours exposure with Methicillin in combination and Alkaloid compounds from*Cienfuegosia digitata*Cav.** Absence of colonies after 6 h. UFC uncountable before 2 h. The results are the means of number of the colonies ± standard deviations.

**Figure 7:**
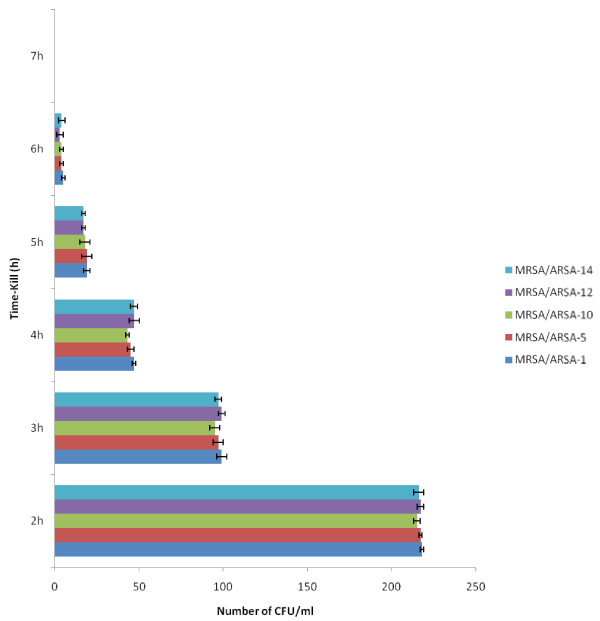
** Viability of microorganisms after 6 hours exposure with Ampicillin in combination and Alkaloid compounds from*Cienfuegosia digitata*Cav.** Absence of colonies after 6 h. UFC uncountable before 2 h. The results are the means of number of the colonies ± standard deviations.

In the micro-well dilution assay (MIC) and Minimum bactericidal concentration (MBC) of alkaloid compounds and their combination with conventional antibiotics (Methicillin and Ampicillin), result varied according to microorganism (Figures 3 and 4). The MIC values were ranged from 12.5 to 50 μg/ml and as for the MBC values were ranged from 25 to 200 μg/ml. The bactericidal and bacteriostatic effect of alkaloid compounds and their combination with conventional antibiotics was determined using the ratio MBC/MIC (Table 1). Concerning the time-kill assay, the results showed that after 6 h exposition there was no viable microorganism in the initial inoculums (Figure 5, 6 and 7).

**Table 1:** **Bacteriostatic (−) and Bactericidal (+) effects of Alkaloid compounds from*Cienfuegosia digitata*Cav. and their combination with Methicillin and with Ampicillin**

**Microorganisms**	**Meth + Alkaloids**	**Amp + Alkaloids**	**Alkaloid Fractions**
***MRSA/ARSA-01***	**+**	**+**	**+**
***MRSA/ARSA-02***	**-**	**-**	**-**
***MRSA/ARSA-03***	**-**	**-**	**-**
***MRSA/ARSA-04***	**+**	**-**	**-**
***MRSA/ARSA-05***	**-**	**+**	**-**
***MRSA/ARSA-06***	**+**	**-**	**-**
***MRSA/ARSA-07***	**-**	**-**	**-**
***MRSA/ARSA-08***	**-**	**-**	**-**
***MRSA/ARSA-09***	**+**	**-**	**-**
***MRSA/ARSA-10***	**-**	**+**	**-**
***MRSA/ARSA-11***	**-**	**-**	**-**
***MRSA/ARSA-12***	**+**	**+**	**+**
***MRSA/ARSA-13***	**-**	**-**	**-**
***MRSA/ARSA-14***	**+**	**+**	**+**
***MRSA/ARSA-15***	**-**	**-**	**-**

The results are the means of number of the colonies ± standard deviations.

**+:** bactericidal effect (MBC/MIC = 1 or 2) –: bacteriostatic effect (MBC/MIC = 4 or 16).

At last, with regard to FICI, our results indicate a synergistic effect between alkaloid compounds and the conventional antibacterial (Tables 2 and 3).

**Table 2:** Fractional Inhibitory Concentration (FIC) and FICI of combination of Alkaloid compounds with Methicillin

**Microorganisms**	**MIC(μg/ml)/Methicillin**	**FIC**_**a**_	**FIC**_**b**_	**FICI**	**Effect**
***MRSA/ARSA-1***	>400	0.50	≤0.03	≤0.53	Synergistic
***MRSA/ARSA-4***	>400	0.50	≤0.03	≤0.53	Synergistic
***MRSA/ARSA-6***	>400	0.25	≤0.03	≤0.28	Synergistic
***MRSA/ARSA-9***	>400	0.25	≤0.03	≤0.28	Synergistic
***MRSA/ARSA-12***	>400	0.5	≤0.03	≤0.53	Synergistic
***MRSA/ARSA-14***	>400	0.5	≤0.03	≤0.53	Synergistic

**FIC**_**a**_ = MIC of the combination/MICa alone; **FIC**_**b**_ = MIC of the combination/MICb alone and FICI = FIC_a_ + FIC_b_.

a = Alkaloid fractions; b = Methicillin.

The FICI was interpreted as follows: (1) a synergistic effect when FICI ≤0.5; (2) an additive or indifferent effect when FICI >0.5 and <1 and (3) an antagonistic effect when FICI >1.

**Table 3:** Fractional Inhibitory Concentration (FIC) and FICI of combination of Alkaloid compounds with Ampicillin

**Microorganisms**	**MIC(μg/ml)/Ampicillin**	**FIC**_**a**_	**FIC**_**b**_	**FICI**	**Effect**
***MRSA/ARSA-1***	>400	0.50	≤0.03	≤0.53	Synergistic
***MRSA/ARSA-5***	>400	0.50	≤0.03	≤0.53	Synergistic
***MRSA/ARSA-10***	>400	0.50	≤0.03	≤0.53	Synergistic
***MRSA/ARSA-12***	>400	0.50	≤0.03	≤0.53	Synergistic
***MRSA/ARSA-14***	>400	0.50	≤0.03	≤0.53	Synergistic

**FIC**_**a**_ = MIC of the combination/MICa alone; **FIC**_**b**_ = MIC of the combination/MICb alone and FICI = FIC_a_ + FIC_b_.

a = Alkaloid fractions; b = Ampicillin.

The FICI was interpreted as follows: (1) a synergistic effect when FICI ≤0.5; (2) an additive or indifferent effect when FICI >0.5 and <1 and (3) an antagonistic effect when FICI >1.

## Discussion

Today, there is a renewed interest in traditional medicine and an increasing demand for more drugs from plant sources. This revival of interest in plant-derived drugs is mainly due to the current widespread belief that green medicine is safe and more dependable than the costly synthetic drugs, many of which have adverse side effects [31]. In effect, herbal remedies used in folk medicine provide an interesting and still largely unexplored source for the creation and development of potentially new drugs for chemotherapy which might help overcome to growing problem of resistance and also the toxicity of the currently available commercial antibiotics [32]. There are a lot of antimicrobial drugs of which some are discovered or established and over 250,000 undiscovered flowering plants with medicinal properties exist in worldwide [33]. Hence, the last decade witnessed an increase in the investigations on plants as a source of human disease management [34] and more natural antimicrobials have driven scientists to investigate the effectiveness of inhibitory compounds such as extracts from plants [35].

Phytochemical constituents such as tannins, flavonoids, alkaloids and several other aromatic compounds are secondary metabolites of plants that serve as defense mechanisms against predation by many microorganisms, insects and herbivores [36]. Several studies have been conducted on the antimicrobial activity of plant extracts found in folk medicine or isolated compounds such as alkaloids. Previous studies have showed that over compounds such as alkaloids have strong antimicrobial activities [17]. The report justifies the traditional use of *Cienfuegosia digitata* Cav. (Malvaceae) in the bacterial infections because of its capacity of alkaloids compounds [14].

The antimicrobial compounds from plants may inhibit microbial growth by different mechanisms than those generally indicate for antimicrobial agents and may have significant clinical value in the treatment of microbial resistance [37]. Thus, alkaloids activity could be attributed to their ability to intercalate DNA [38].

In generally, Gram-positive bacteria should be more susceptible since they have only an outer peptidoclycans layer which is not an effective permeability barrier as reported by [39]. But in this study, we found contradicting results. *Staphylococcus aureus* some Gram-positive has developed resistance to the β-lactam antibiotics due to the production of chromosomal or plasmid mediated β-lactamases or by producing penicillin binding proteins (PBPs). All the *Staphylococcus aureus* strains have from PBPs (PBP1 to PBP4), but MRSA express a special PBP (PBP2 or PBP2a) from the mec A gene PBP2a takes over the biosynthetic function of normal PBPs in the presence of inhibitory concentration of β-lactams because PBP2 has a decreased binding affinity to β-lactams [40]. This has resulted in the development of multidrug resistance against β-lactam and other antibiotics. In addition, the polysaccharide capsular material in some of the pathogenic microorganism is responsible for virulence and antimicrobial resistance [41]. This may also explain another aspect of bacterial resistance to conventional antibiotics. However one note that the alkaloid compounds or their combination with conventional antibiotics have effects on bacteria. This could be explained by the fact that the alkaloid compounds inhibit or destroy the action of β-lactamase.

Indeed, synergy research in phytomedicine has established itself as a new key activity in recent years. It is one main aim of this research to find a scientific rational for the therapeutic superiority of herbal drugs derived from traditional medicine as compared with single constituents thereof. Synergy effects of the mixture of bioactive constituents and their byproducts contained in plant extracts are claimed to be responsible for the improved effectiveness of many extracts and conventional antimicrobial drugs [28].

## Conclusion

The results of present study supports the traditional usage of *Cienfuegosia digitata* Cav. (Malvaceae) and suggests that this herbaceous possess compounds with high antibacterial properties that can be used as antibacterial agents in developing new drugs for the therapy of infectious diseases caused by pathogenic bacteria. Moreover, based on the results of this study, alkaloid compounds of *Cienfuegosia digitata* Cav. (Malvaceae) should be analyzed further because of their potential as a source of broad spectrum antimicrobial compounds which can be use for treating infectious diseases caused by MRSA/ARSA. This highlights the continuous interest in laboratory screening of medicinal plants, not only to determine the scientific rationale for their usage, but also to discover new active principles.

## References

[B1] Sanches IS Saraiva ZC Tendeir TC Serra JM Dias DC Delencastre H Extensive intra-hospital spread of methicillin resistant Staphyloccocalclone Int J Infect Disease 1998 3 26 31 9831672 10.1016/s1201-9712(98)90091-1

[B2] Bachi BB Rohrer S Factors influencing methicillin resistance in Staphylococci Arch 2002 178 165 171 10.1007/s00203-002-0436-0 12189417

[B3] Hiramatsu K Hamaki H Ito T Yabuta K Ogura T Tenova FC Methicillin resistant Staphylococcus aureus clinical strains with reduced vomcomycin susceptibility J Anti-Microbial Chemother 1997 41 135 146 10.1093/jac/40.1.135 9249217

[B4] Parekh J Chanda S In vitro antibacterial activity of the crude methanol extract of Woodfordia Fructicosa Kurz. Flower (Lythaceae) Braz J 2007 38 204 207

[B5] Dash BK Sultana S Sultana N Antibacterial activities of methanol and acetone extracts of Ferrugreek (Trigonelle foenum) and Coriander (Coriandrum sativum) Life Sci Med Res 2011 27 1 8

[B6] Arya V Yadav S Kumar S Yadav JP Antimicrobial activity of Cassia occidentalis (Leaf) against various human pathogenic microbes Life Sci Med Res 2010 9 1 11

[B7] Barbour EK Al-Sharit M Sagherian VK Habe AN Talhouk RS Talkouk SN Screening of select indigenous plants of Lebanon for antimicrobial activity J Etnopharmacol 2004 93 1 7 10.1016/j.jep.2004.02.027 15182897

[B8] Geyid A Abebe D Debella A Makonnen Z Aberra F Screening of some medicinal plants of Ethiopia for their anti-microbial properties and chemical profiles J Ethnopharmacol 2005 97 421 427 15740876 10.1016/j.jep.2004.08.021

[B9] Rahman MS Rahman MZ Wahab MA Chowdhury R Rashid MA Antimicrobial activity of some indigenous plants of Bangladesh Dhaka Univ J Pharma Sci 2008 7 23 26

[B10] Elvin-Lewis M Should we be concerned about herbal remedies J 2001 75 141 164 10.1016/s0378-8741(00)00394-9 11297844

[B11] Silver L Bostian K Screening of natural products for antimicrobial agents Eur 1990 9 455 461 10.1007/BF01964283 2226472

[B12] Ellof JN Which extractant should be used for the screening and isolation of antimicrobial components from plants J 1998 60 1 8 10.1016/s0378-8741(97)00123-2 9533426

[B13] Karim A Nouman M Munir S Sattar S Pharmacology and phytochemistry of Pakistani herbs and herbal drugs used for treatement of diabetes Int J Pharmacol 2011 7 419 439

[B14] Nacoulma OG Medicinal plants and their traditional uses in Burkina Faso 1996 University of Ouagadougou, Ph.D.Thesis 328

[B15] Konaté K Souza A Polyphenol Contents, Antioxidant and Anti-Inflammatory Activities of Six Malvaceae Species Traditionally used to treat Hepatitis B in Burkina Faso Eur J Sci Res 2010 44 4 570 580

[B16] Le Minor L Veron M Bactériologie Médicale, eds Flammarion medecine-sciences 1984 773 ISBN 2-257-10418-8

[B17] Karou D Savadogo A Canini A Yameogo S Montesano C Simpore J Colizzi V Traore AS Antibacterial activity of alkaloids from Sida acuta Afr 2006 5 2 195 200

[B18] Perez C Pauli M Bazerque P An antibiotic assay by the agar-well diffusion method Acta Biologiae et Medecine Experimentalis 1990 15 113 115

[B19] Ezoubeiri A Gadhi CA Fdil N Benharref A Jana M Vanhaelen M Isolation and antimicrobial activity of two phenolic compounds from Pulicaria odora L J 2005 99 287 292 10.1016/j.jep.2005.02.015 15894140

[B20] Pujol V Villard J Research of antifungal substances secreted by higher fungi in culture French Pharmaceut J 1990 48 17 22 2082797

[B21] Rabe T Mullholland D Van Staden J Isolation and identification of antibacterial compounds from Vernonia colorata leaves J 2002 80 91 94 10.1016/s0378-8741(02)00010-7 11891091

[B22] Performance standard for anti-microbial susceptibilitytesting:eleventh informational supplement. Document M100-S11 2001 National Committee for Clinical Laboratory Standard, Wayne, PA, USA

[B23] Traoré R Contribution has the Study of the Adhesion of Enterobacteries of the Kinds Klebsiella proteus and K serratia with the Human epithelial Cells. Doct thesis science pharmaceutical 1993 Université libre de Bruxelles 158

[B24] Berche P Gaillard JL Simonet M Nosocomial Infections Caused by Bacteria and Their Prevention in Bacteriology Ed Flammarion Medicine Sciences 1988 64 71

[B25] Wolfe EF Klepser ME Pfaller MA Antifungal dynamics of amphotericin B and fluconazole in combination against Candida albicans, effect of exposure time Pharmacotherapy 1997 17 189 189

[B26] White RL Bugess DS Manduru M Bosso JA Comparison of three different in vitro methods of detecting synergy: Time-kill, chercherboard and E test Antimicrob 1996 40 1914 1918 10.1128/aac.40.8.1914 PMC163439 8843303

[B27] Methods for Determining Bactericidal Activity of Antimicrobial Agent 1992 National Committee for Clinacal Laboratory Standards, Wayne, Pa

[B28] Rosato A De Laurentis NVN Armenise D Millilo MA Antibacterial effect of some essential oils administered alone or in combination with Norfloxacin Phytomedicine 2007 14 727 732 17303397 10.1016/j.phymed.2007.01.005

[B29] Wolfe EF Klepser ME Pfaller MA Antifungal dynamics of amphotericin B and fluconazole in combination against Candida albicans, effect of exposure time Pharmacotherapy 1997 17 189 189

[B30] Williamson EM Synergy and other interactions in phytomedicines Phytomedicine 2001 8 401 409 11695885 10.1078/0944-7113-00060

[B31] Agbafor KN Akubuguo EI Ogbashi ME Ajah PM Ukwandu CC Chemical and antimicrobial properties of leaf extracts of Zapoteca portoriceusis Res J Med Plant 2011 5 605 612

[B32] Alam MT Karim MM Kham SN Antibacterial activity of different organic extracts of Achyranthes aspera and Cassia alata J Sci Res 2009 1 393 398

[B33] Madureira MDC Rediscovering traditional medicine spore 2008 136 16 17

[B34] Woldemichael GM Wachter G Singh MP Maiese WM Timmermann BN Antibacterial diterpene from Calceolaria pinifolia J Nat Prod 2003 66 242 246 12608857 10.1021/np020380x

[B35] Nasar-Abbas SM Halkman AK Antimicrobial effect of water extract of sumac (Rhus coriaria L.) on the growth of some food borne bacteria including pathogens Int J Food Microbiol 2004 97 63 69 15527919 10.1016/j.ijfoodmicro.2004.04.009

[B36] Doughari JH Okafor NB Antibacterial activity of Senna siamae leaf extracts on Salmonella typhi Afr J Microbiol Res 2008 2 42 46

[B37] Ellof JN Which extractant should be used for the screening and isolation of antimicrobial components from plants J Ethnopharmacol 1998 60 1 8 9533426 10.1016/s0378-8741(97)00123-2

[B38] Cowan MM Plants products as antimicrobial agents Clin Microbiol Rev 1999 12 564 582 10515903 10.1128/cmr.12.4.564 PMC88925

[B39] Nostro A Germano MP D’Angelo V Marino A Cannatelli MA Extraction methods and bioautography for evaluation of medicinal plant antimicrobial activity Lett. Applied Microbial 2000 30 379 384 10.1046/j.1472-765x.2000.00731.x 10792667

[B40] Bachi BB Rothrer S Factors influencing methicillin resistance in Staphylococci Arch Microbiol 2002 178 165 171 12189417 10.1007/s00203-002-0436-0

[B41] Hooper DC Emerging mechanisms of fluoroquinolone resistance Emerg Infect Dis 2001 7 337 341 11294736 10.3201/eid0702.010239 PMC2631735

